# Complete genome sequence of a virulent duck enteritis virus isolated from Northern part of Bangladesh

**DOI:** 10.1128/mra.00774-25

**Published:** 2025-10-24

**Authors:** Layla Yasmin, Md. Shahrior Siddique, Towhidul Islam, Mohammad H. Rahman, Md. Hasibur Rahman, Shuponkor Ghosh, Mohammad Ferdousur Rahman Khan, Marzia Rahman, Md. Bahanur Rahman

**Affiliations:** 1 Department of Microbiology and Hygiene, Bangladesh Agricultural University 54492, Mymensingh, Bangladesh; 2 Tamim Agro Industries Ltd., Bogura, Bangladesh; 3 Department of Biotechnology, Bangladesh Agricultural University 54492, Mymensingh, Bangladesh; Katholieke Universiteit Leuven, Leuven, Belgium

**Keywords:** complete genome, DEV, GC content, genome size, Illumina, NextSeq 2000

## Abstract

This study reports the complete genome sequencing of the duck enteritis virus strain YLBRDP_11 isolated from liver tissue of ducks during a local outbreak in Netrokona district, Northern part of Bangladesh. The viral genome consists of 161,633 bp encoding 74 proteins with a GC content of 44.9%.

## ANNOUNCEMENT

Duck viral enteritis (DVE), commonly known as Duck plague, is a severe and highly contagious disease of waterfowl, including ducks, geese, and swans (1). DEV is a member of the *Orthohervisviridae* family, subfamily *Alphaherpesvirinae*, genus *Mardivirus,* species *Mardivirus anatidalpha1,* also known as Anatid herpesvirus 1 (2). It is an enveloped virus with linear double-stranded DNA (dsDNA) genome consisting of 158,091–162,175 bp (3–5).

Duck plague is documented worldwide, including Bangladesh, and causes significant economic impact due to its high morbidity and fatality rates (6). During a natural outbreak in Netrokona district (24°47″ to 24°58″ N and 90°38″ to 90°50″E) of Bangladesh in 2023, we collected liver samples from dead ducks and preserved them at −80°C until further processing. Homogenization of liver samples was performed to prepare a 10% suspension in 1× phosphate-buffered saline and centrifuged at 4,000 rpm for 10 min (1). The supernatant was treated with gentamicin for sterility testing. Sterile samples were used for DNA extraction employing QIAamp DNA Mini Kit (7) (QIAGEN, Germany) as per the instructions of manufacturer and confirmation by PCR for DNA polymerase gene (446 bp) (8). The DEV-positive sample was propagated in 11-day-old embryonated duck eggs (EDEs) via chorioallantoic membrane (CAM) route up to 10th passage (9), followed by pathogenicity test in adult ducks to assess the virulence of the virus (8).

Genomic DNA was extracted from the DEV-positive CAM of EDE using the previously mentioned kit. DNA concentration and quality were evaluated by NanoDrop One (Thermo Scientific, Canada) (10). A DNA library was constructed by NEBNext Ultra II DNA library prep kit for Illumina (NEB, USA) and sequenced on the NextSeq 2000 system, producing 2 × 150 bp paired-end reads at the Genomics Laboratory, Child Health Research Foundation, Bangladesh (11). The sequencing run produced 45,447,254 reads exhibiting an average quality score of Q33, indicating a base call accuracy of 99.95%, assessed by BUSCO v5.12 (12).

Genome assembly and annotation were performed using Geneious Prime (v2025.1.3). The raw FASTQ read files were imported as paired read sets. The reads underwent quality trimming and filtering via BBDuk (BBDuk Adapter/Quality Trimming v38.84) plugin of Geneious Prime, where the adapters and low-quality (30 nt) reads at both ends were trimmed, additionally discarding short reads (<30 nt) (13, 14). After trimming the data set, error correction and normalization were done by BBNorm v38.84 (15). Genome assembly was performed by mapping normalized reads to reference genome of CHv strain of Anatid alphaherpesvirus 1 (GenBank assembly number GCA_027935785.1). Reads were mapped using Geneious algorithm (medium-low sensitivity/fast settings), results were saved as consensus sequence used as the final genome.

**Fig 1:**
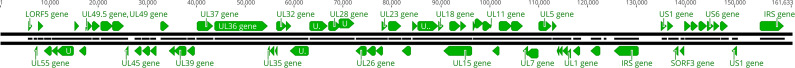
Genomic map with labeled open reading frames and genes including LORF5, UL49.5, UL36, UL11, UL1, and IRS, arranged bidirectionally along nucleotide positions from 0 to 161,633.

Out of 45,447,254 generated reads, 37,155,624 were used for alignment. Using Geneious Prime (v2025.1.3), 421,887 normalized reads were aligned to the reference genome (GCA_027935785.1), generating a 161,633 bp consensus sequence covering 99.67% (161,633 of 162,175 bp) of the genome (Fig. 1). The complete genome of YLBRDP_11 spans 161,633 bp, encoding 74 genes, GC content of 44.9%. BLASTn analysis exhibited up to 99.99% nucleotide similarity with other DEV isolates in the NCBI database (16).

## Data Availability

The YLBRDP_11 genomic sequence has been archived in GenBank under the accession number PV612389, and the SRA project data are available under the SRA accession number SRR32944910. BioProject and BioSample ID are PRJNA1245142 and SAMN47742526, respectively.
